# Macrophage migration inhibitory factor levels are associated with disease activity and possible complications in membranous nephropathy

**DOI:** 10.1038/s41598-022-23440-1

**Published:** 2022-11-03

**Authors:** Na Ding, Peng-Lei Li, Kai-Li Wu, Tie-Gang Lv, Wen-Lu Yu, Jian Hao

**Affiliations:** 1 Renal Division, Department of Medicine, The Affiliated Hospital of Inner Mongolia Medical University, Huhehot, 010050 Inner Mongolia China; 2 Inner Mongolia Medical University, Huhehot, 010059 Inner Mongolia China

**Keywords:** Diseases, Nephrology, Risk factors

## Abstract

Membranous nephropathy (MN) is an autoimmune disease characterized by the deposition of immunoglobulin G (IgG) and complementary components in the epithelium of the glomerular capillary wall. Macrophage migration inhibitory factor (MIF) is an inflammatory mediator released by macrophages. MIF plays a key regulatory function in the pathogenesis of immune-mediated glomerulonephritis. This study aimed to investigate whether MIF level could be associated with the activity of MN. Plasma and urine samples from 57 MN patients and 20 healthy controls were collected. The MIF levels in plasma and urine were determined by an enzyme-linked immunosorbent assay (ELISA) kit. The expression of MIF in the renal specimens from 5 MN patients was detected by immunohistochemistry (IHC). The associations of the plasma and urinary levels of MIF and glomerular MIF expression with clinical and pathological characteristics were analyzed. It was revealed that with the increase of MIF levels in plasma and urine, the severity of renal pathological injury in MN patients gradually increased. Correlation analysis showed that the MIF levels in plasma were positively correlated with the platelet (PLT) count (r = 0.302, *P* = 0.022), and inversely correlated with the prothrombin time (PT) (r =  − 0.292, *P* = 0.028) in MN patients. The MIF levels in plasma were positively correlated with the C-reactive protein (CRP) level and erythrocyte sedimentation rate (ESR) (r = 0.651, *P* < 0.0001; r = 0.669, *P* < 0.0001) in MN patients. The urinary levels of MIF were positively correlated with ESR (r = 0.562, *P* < 0.0001). IHC suggested that MIF was expressed in glomerular basement membrane and tubulointerstitial areas. MIF levels in plasma and urine could reflect the severity of MN, and MIF levels in plasma and urine could be associated with venous thrombosis and infectious complications in MN patients. The glomerular MIF expression could be used to indicate the activity of MN.

## Introduction

Membranous nephropathy (MN) is a glomerular disease, in which immune deposits of immunoglobulin G (IgG) and complement components develop predominantly or exclusively beneath podocytes on the subepithelial surface of the glomerular capillary wall^1^. MN occurs in all regions and all ethnicities with an annual incidence of 10–12 per million in North America and 2–17 per million in Europe and it is a leading cause of nephrotic syndrome (NS) in adults^2^. With the exploration of podocyte antigens and associated autoantibodies, MN can be classified into three categories based on antigen specificity. M-type phospholipase A2 receptor (PLA2R) and thrombospondin type-1 domain-containing 7A (THSD7A) are known as target podocyte antigens in MN^3,4^. When the target antigen is unknown and the MN is irrelevant to any other known systemic diseases or secondary cause, idiopathic MN is diagnosed. If the kidney damage would be related to other systemic diseases, such as infection, autoimmune diseases, malignancy, etc., secondary MN is diagnosed. The pathogenesis of MN is complicated and, and if it remains untreated, it presents as a unique clinical process, in which, as previously reported, one-third of patients achieved complete remission, on-third had partial remission, and the remaining progressed to the end-stage renal disease (ESRD)^5^.

The macrophage migration inhibitory factor (MIF), a protein of 115 amino acids, is expressed in a variety of organs and cell types, such as macrophages, monocytes, epithelial cells, and platelets^6,7^. Binding of MIF to its cell membrane receptor CD74 leads to recruitment of the cell-surface glycoprotein CD44^8,9^. In addition, CD74 is coupled with CD44 to initiate a signaling cascade, leading to the subsequent expressions of pro-inflammatory cytokines. MIF binds to CXC chemokine receptor types 2, 4, and 7, attracting macrophages and directing B lymphocytes to the site of inflammation, and MIF also indirectly boosts the immune response by inhibiting the glucocorticoid actions^10^.

To date, a large number of experimental studies have found that MIF is an essential signaling cytokine in the immune system and it binding to its molecular targets plays a key role in inflammatory processes^11^. MIF can attract macrophages and T lymphocytes to infiltrate and accumulate in inflammation and enhance their phagocytic function, inhibit their migration, and promote the proliferation, activation, and secretion of certain cytokines, thereby mediating kidney disease. A previous study found that a small-molecule inhibitor of MIF protects lupus-prone mice from kidney disease^12^. MIF acts through several mechanisms to mediate renal injury, inhibiting movement of macrophages to other sites, as well as promoting the proliferation, activation, and secretion of some cytokines. Additionally, MIF can stimulate kidney podocytes to secrete proinflammatory factors, which can accelerate glomerulosclerosis and eventually lead to an irreversible damage to the kidney. A previous research found that anti-MIF treatment can reduce macrophage aggregation in kidney tissue, ameliorate kidney failure, and cause delay in the reduction of renal function^13^.

However, no association between MIF levels and the activity of MN was reported. The current study aimed to investigate whether MIF level could be associated with the activity of MN.

## Methods

### Patients and samples

In total, 57 patients with MN diagnosed at Affiliated Hospital of Inner Mongolia Medical University from November 2019 to October 2020, were enrolled in our study. Plasma and urine samples from these patients were collected before the initiation of hormone and immunosuppressive treatment. In addition, we collected plasma and urine samples from 20 healthy blood donors, respectively, as normal controls. This study excluded patients with severe infections (especially urinary infections), renal biopsy findings incorporating other types of glomerular nephritis (such as lupus nephritis, LN, IgA kidney disease), other autoimmune diseases (such as Graves disease, rheumatoid arthritis, etc.) and MN caused by systemic diseases such as infection, autoimmune diseases, malignancy, drugs or injury exposure factors.Before renal puncture, a volume of 5 mL venous blood from each patient was withdrawn and collected in EDTA anticoagulation tubes. The supernatant was separated by centrifugation at 3000 rpm, 5 min, and stored at − 80 °C for subsequent use. Urine samples of all patients and healthy blood donors were frozen directly in the ep tube within 10 min in the − 80 °C refrigerator. Repeated freeze/thaw cycles were avoided. Kidney tissue specimens were collected in 5 of the above 57 MN patients. Six renal tissues were obtained from the normal part of nephrectomized (because of renal carcinoma) kidneys and were used as normal controls; they were determined to be normal using light microscopy, immunofluorescence and electron microscopy. Written informed consent was obtained from each participant. We collected baseline patient data including age, gender, kidney history, positive signs, disease complications etc.Before renal puncture, registration (Urine protein quantification/24 h, UTP/24 h), Creatinine (Cr), serum albumin (ALB), PLA2R-Ab, C-reactive protein (CRP), Blood Down (ESR), fibrinogen (FIB), Prothrombin Time (PT), activated partial prothrombin time (APTT), thrombin time (TT), fibrin (primary) degradation products (FDP), D-dimer, triglyceride (TG), total cholesterol (TCH), low-density lipoprotein (LDL-C), as well as high-density lipoprotein (HDL-C) and other clinical indicators. All patients provided their informed written consent. All the methods were performed in accordance with relevant guidelines and regulations. All methods were approved by the Helsinki Declaration of Inner Mongolia Medical University Ethics committee.

### Detection of MIF by enzyme-linked immunosorbent assay (ELISA)

Plasma and urine MIF concentrations were analyzed by ELISA using commercial kits (Bioss Biotechnology Co, Beijing, China) according to the manufacturers’ instructions and by comparison to the standard curve.

### Renal histology

Renal histology of MN patients was evaluated according to Ehrenreich-Churg standards^14^. The presence of glomerular lesions, including glomerular sclerosis, crescent moon, and segment sclerosis, were calculated as a percentage of the total number of glomeruli in a biopsy. Tubular and interstitial lesions were scored semi-quantitatively on the basis of the percentage of the tubulointerstitial compartment that was affected: the tubular atrophy(“−” for 0%, “+” for 0% − 50%, “++” for > 50%), interstitial fibrosis (“−” for 0% , “+” for 0% − 50%, “++” for > 50%) and interstitial infiltration (“−” for 0%, “+” for 0% − 20%, “++” for 20% − 50%, “+++” for > 50%).

### Detection of MIF expression in kidneys by immunohistochemistry

The specimens were deparaffinized overnight before staining. They were then further deparaffinized in xylene and rehydrated through graded ethanol. Antigen retrieval was performed by heating the slides in citrate buffer (pH 6.0) in an 200 W microwave oven for 40 min. The slides were then cooled to room temperature and washed in phosphate buffered saline (PBS). Endogenous peroxidase activity was quenched with 3% hydrogen peroxide at room temperature for 10 min. Non-specific staining was blocked by incubating the specimens with 3% bovine serum albumin (BSA) in PBS at 37 °C for 30 min. After removing BSA without washing, primary antibodies were added (anti human MIF, Abcam, Cambridge, UK) and then incubated at 4 °C pass the night. Secondary antibodies (MXB, Fu zhou, China) were incubated with the specimens at 37 °C for 10 min. Next, the specimens were developed in streptomyces anti-biotin protein-peroxidase for at 37 °C for 10 min. Eventually the specimens were incubated with haematoxylin and then dehydrated through graded alcohol and xylene. The expression of each MIF was observed under a light microscope. We used the Image-Pro Plus analysis software (version 6.0; Media Cybernetics, Dallas, TX, USA) to evaluate the renal staining of MIF. Positive signals were quantified as the mean optical density (integrated option density/area). All the glomeruli in a section at × 400 were observed blindly as a semiquantitive assessment of renal immunohistochemical staining.

### Statistical analysis

All data were statistically analyzed using SPSS22.0. Quantitative data were expressed as means ± SD (for data that were normally distributed) or median and range (for data that were not normally distributed). The two groups of enumeration data that conformed to the normal distribution were compared using an independent t test. If any of the groups did not match, a non-parametric rank-sum test was used. For two parametric variables, correlation analysis between two continuous variables was performed using Pearson’s correlation. For two nonparametric variables or one non-parametric variable with one parametric variable, correlation analysis between two continuous variables was performed using Spearman’s rank correlation. If *P* < 0.05, differences were considered statistically significant.

## Results

### MN patients’ clinical data

Of the 57 patients with MN, 34 (59.65%) cases were male and 23 (40.35%) cases were female, with an average age of 46.96 ± 14.07 years old at the time of diagnosis. Besides, 35 patients were PLA2R-Ab-positive; –22 patients were PLA2R-Ab-negative. The range of serum creatinine (Cr) level was 44–232 μmol/L. The range of the 24-h urine protein test (UTP) was 1.13–15.8 g/24 h. Among 21 patients who developed eyelid and facial edema, 49 patients developed limb edema, and 8 patients showed no obvious signs.

Compared with group MN, white blood cell (WBC) count and levels of fibrinogen (FIB), erythrocyte sedimentation rate (ESR), triglyceride (TG), total cholesterol (TCH), and low-density lipoprotein-cholesterol (LDL-C) were significantly elevated in the control group; however, the levels of albumin (ALB and HDL-C were significantly reduced. There were no statistically significant differences in age and gender between MN and control groups. The patients’ general clinical data and diagnostic indicators are presented in Tables 1 and 2, respectively.

**Table 1:** General data for patients with MN.

General data	Quantity (%)
Amount	57
Gender (male/female)	34/23
Age	46.96 ± 14.07
Eyelid and facial edema	21 (36.84%)
Limb edema	49 (85.96%)
Blood creatinine levels (umol/L)	57 (49.50, 79.75)
Urinary protein quantification (g/24 h)	4.93 (2.8, 8.04)

**Table 2:** Comparison of clinical indicators in MN and control.

Clinical indicators	MN (n = 57)	Control (n = 20)	*P*
HGB (g/L)	138.5 ± 15.63	136.7 ± 12.04	0.643
WBC (10^9^/L)	5.87 (5.34, 6.91)	5.34 (4.59, 6.02)	0.017
PLT (10^9^/L)	239.6 ± 58.15	211.4 ± 47.05	0.054
ALB (g/L)	26.46 ± 7.56	39.49 ± 4.36	< 0.0001
PT (s)	10.25 ± 0.68	10.14 ± 0.70	0.547
APTT (s)	24.80 (24.20, 25.90)	24.60 (24.2, 25.55)	0.749
TT (s)	17.6 (16.8, 18.2)	17.15 (15.93, 18.13)	0.241
FIB (g/L)	4.29 (3.38, 5.06)	3.29 (2.85, 3.44)	< 0.0001
FDP (ug/ml)	1.85 (1.06, 3.30)	2.05 (1.06, 3.18)	0.984
D-dimer (ug/ml)	0.37 (0.23, 1.01)	0.33 (0.23, 0.42)	0.232
CRP (mg/L)	1.61 (0.50, 2.69)	0.52 (0.50, 1.21)	0.002
ESR (mm/h)	33 (19, 53.5)	8 (6.25, 11.75)	< 0.0001
TG (mmol/L)	2.07 (1.28, 5.47)	0.58 (0.33, 0.74)	< 0.0001
TCH (mmol/L)	6.28 ± 2.82	3.93 ± 0.77	0.0005
HDL-C (mmol/L)	1.16 (0.88, 1.39)	1.71 (1.62, 1.77)	< 0.0001
LDL-C (mmol/L)	3.75 (2.29, 6.20)	2.30 (1.80, 2.62)	0.0002

### MIF level could reflect the severity of MN, and it had no correlation with PLA2R-Ab expression

**Figure 1:**
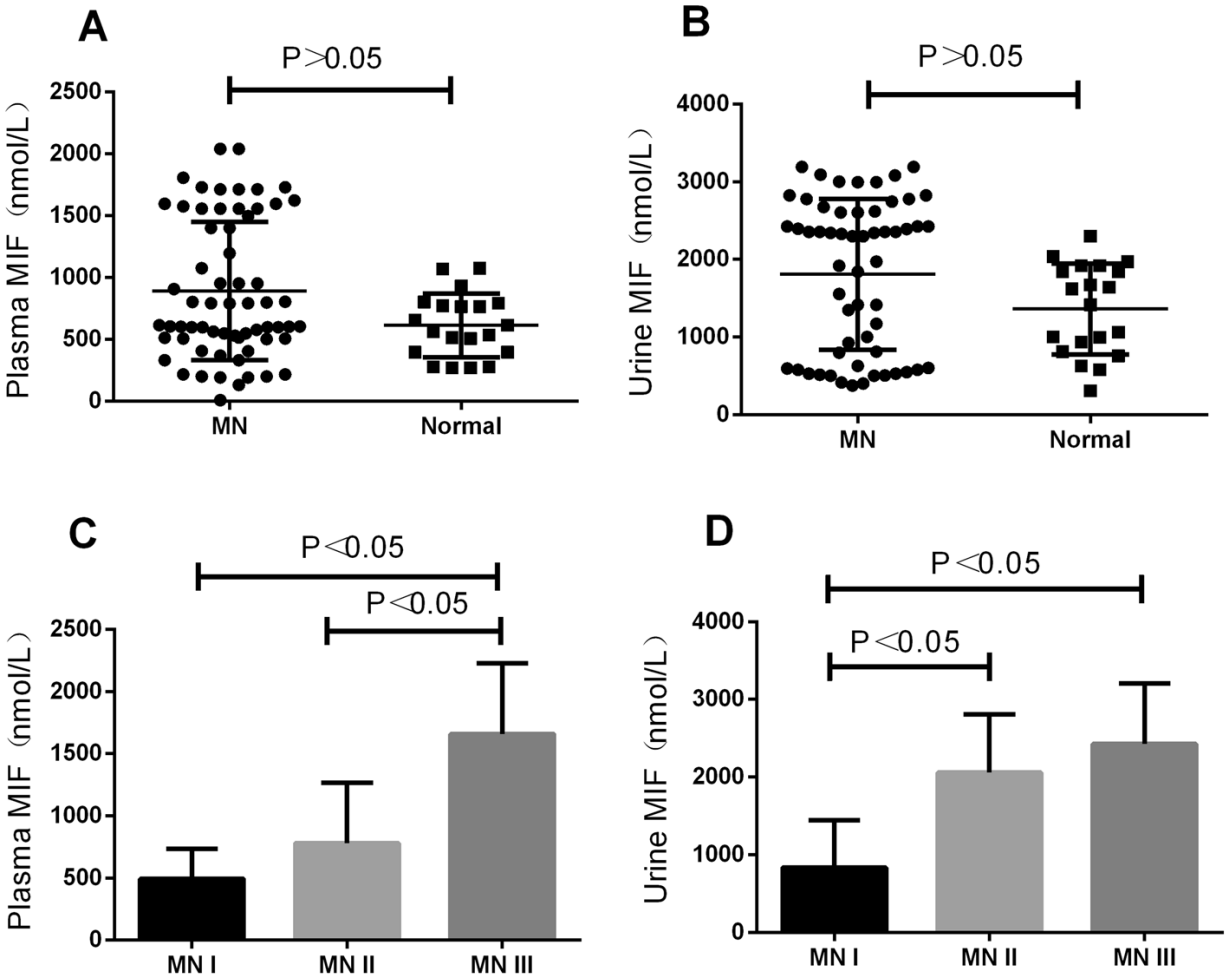
(**A**) Patients with MN (n = 57) were compared with plasma MIF levels in healthy controls (n = 20). (**B**) Patients with MN (n = 57) were compared with urinary MIF levels in healthy controls (n = 20). (**C**) Comparison of plasma MIF levels in patients with different MN. (**D**) Comparison of urinary MIF levels in patients with different MN.

Plasma and urine samples were collected from 57 MN patients and 20 healthy blood donors to detect MIF levels in the plasma and urine using a commercial enzyme-linked immunosorbent assay (ELISA) kit. It was found that there were no statistically significant differences in the MIF levels in plasma and urine between MN and control groups (*P* > 0.05) (Fig. 1A,B).

To compare the differences in MIF levels in plasma and urine between patients with different pathological stages of MN, we pathologically divided all MN patients into 3 groups (MN-I (n = 17), MN-II (n = 22), and MN-III (n = 18)). Further comparing the differences in MIF levels in plasma and urine among MN patients by pathological staging showed that, with the increased severity of the renal pathology, MIF levels in plasma gradually increased, and MIF levels in plasma in MN-III group were higher than those in MN-II group (*P* < 0.05); MIF levels in plasma in MN-III group were higher than those in MN-I group (*P* < 0.05), and the differences were signification (Fig. 1C). Additionally, it was revealed that with the increased severity of the renal pathology, the MIF levels in urine gradually increased, and urinary MIF levels in MN-III group were higher than those in MN-I group (*P* < 0.05); urinary MIF levels in MN-II group were higher than those in MN-I group (*P* < 0.05), and the differences were statistically significant (Fig. 1D).

**Figure 2:**
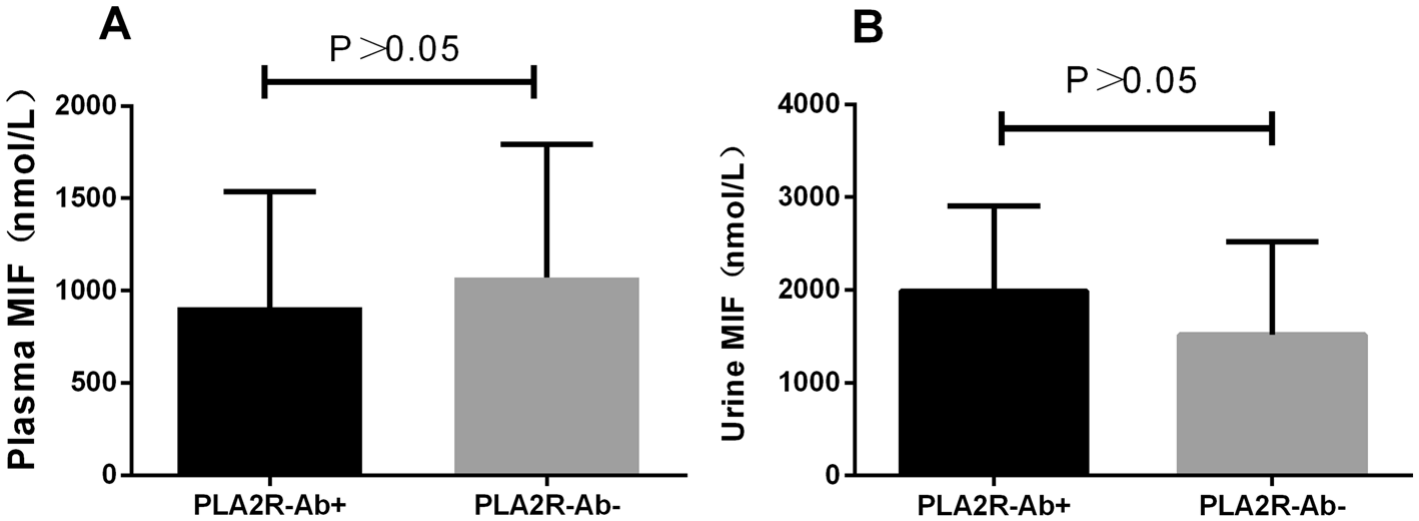
(**A**) Comparison of PLA2R-Ab-positive (n = 35) versus PLA2R-Ab-negative (n = 22) plasma MIF levels in patients with MN. (**B**) Comparison of PLA2R-Ab positive (n = 35) versus PLA2R-Ab negative (n = 22) urine MIF levels in patients with MN.

According to the comparison of PLA2R-Ab-positive (n = 35) and PLA2R-Ab-negative (n = 22) groups, there was no significant difference in MIF levels in plasma (*P* > 0.05) (Fig. 2A). The comparison of PLA2R-Ab-positive (n = 35) and PLA2R-Ab-negative (n = 22) groups showed that there was no significant difference in urinary MIF levels (*P* > 0.05) (Fig. 2B).

### The association of MIF levels with infection and the occurrence of venous thrombosis

**Figure 3:**
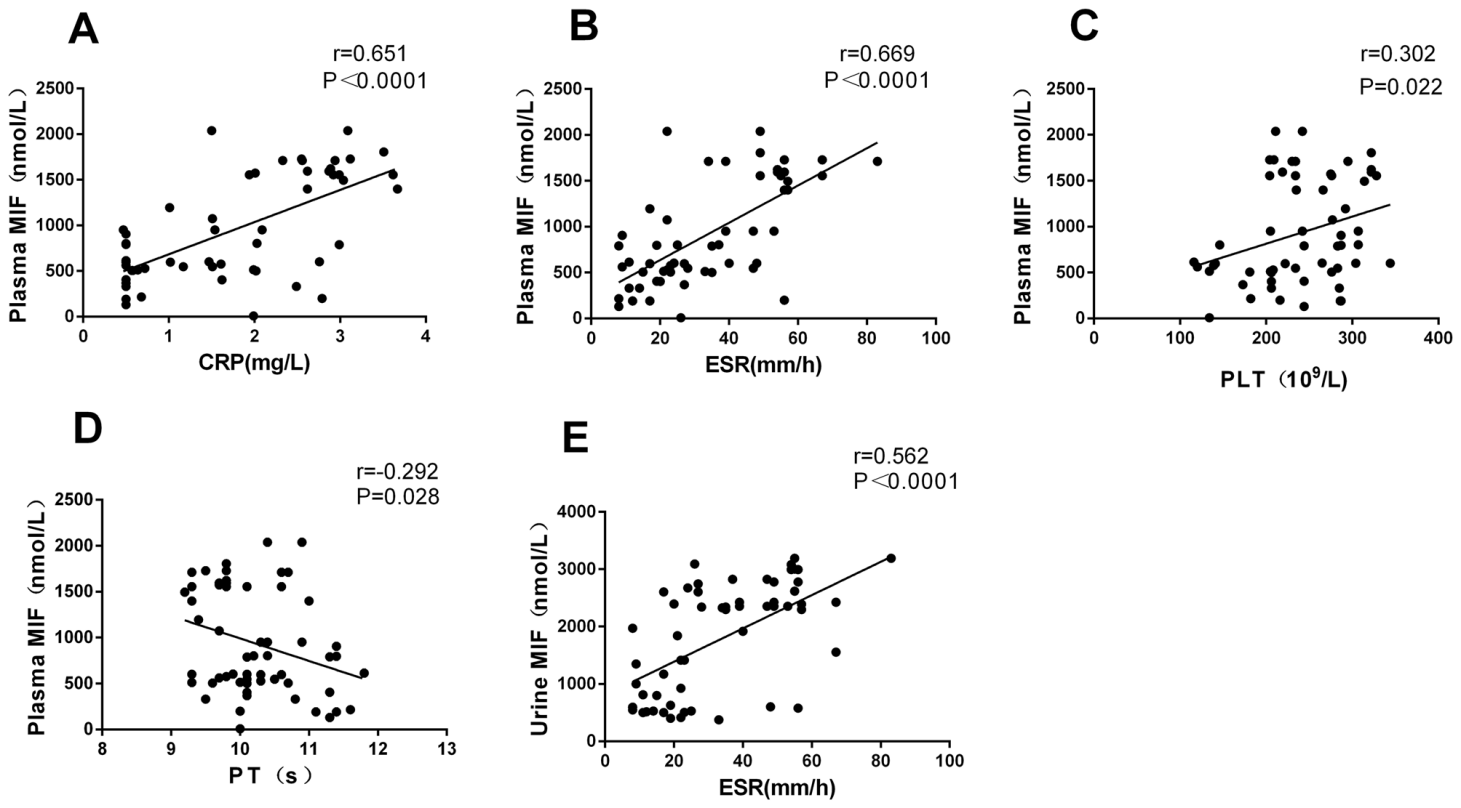
Comparison of plasma and urinary MIF levels with infection (CRP, ESR) and coagulation (PLT, PT). (**A**) The plasma MIF level was positively associated with CRP levels (*P* < 0.0001, r = 0.651). (**B**) The plasma MIF levels were positively associated with ESR levels (*P* < 0.0001, r = 0.669). (**C**) The plasma MIF levels were positively associated with PLT levels (*P* = 0.022, r = 0.302). (**D**) The plasma MIF levels were negatively associated with PT levels (*P* = 0.028, r =  − 0.292). (**E**) Urinary MIF levels were positively associated with ESR levels (*P* < 0.0001, r = 0.562).

The relationship between MIF levels in plasma with inflammatory coagulation in MN patients was analyzed. The results showed that MIF levels in plasma were positively correlated with CRP and ESR indicators (r = 0.651, *P* < 0.0001; r = 0.669, *P* < 0.0001) (Fig. 3A, B). Besides, MIF levels in plasma were positively correlated with PLT (r = 0.302, *P* = 0.022) and negatively correlated with PT (r =  − 0.292, *P* = 0.028) (Fig. 3C, D), while no association of MIF levels with APTT, TT, FDP, FIB, and D-dimer was found. The above-mentioned results suggested that plasma MIF levels in MN patients could be associated with infection and complications of venous thrombosis.

The urinary MIF levels were also compared with inflammatory coagulation index in MN patients. It was found that urinary MIF levels were positively correlated with the ESR (r = 0.562, *P* < 0.0001) (Fig. 3E), while no correlation of urinary MIF levels with PLT, PT, TT, FIB, FDP, and D-dimer was noted. These results suggested that urinary MIF concentrations in MN patients could be associated with infectious complications.

### MIF was significantly expressed in the renal tissues of MN patients and was associated with 24-h UTP

Of the 57 patients, renal tissues of 5 MN patients were collected, including 3 in MN-I group, 2 in MN-II group, and these 5 MN patients were of PLA2R-Ab-negative. Among these 5 patients, there were 3 men (60%) and 2 women (40%), and the general pathology of MN patients is presented in Table 3.

**Table 3:** General pathological data for patients with MN.

Pathological indicators	Quantity (%)
Glomerular lesions	
Scale of glomerular sclerosis (%)	5(0, 10.97)
Small tubule interstitial damage	
The tubules atrophy (−/+/++)	1/4/0
Interstitial fibrosis (−/+/++)	1/4/0
Interstitial infiltration (−/+/++/+++)	1/1/3/0

**Figure 4:**
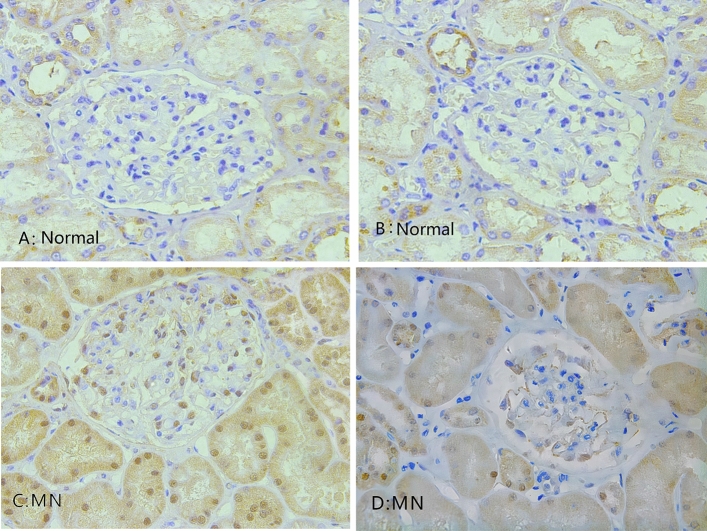
Expression of MIF in renal tissue (light microscope × 400). (**A**,**B**) MIF expression in the control group. (**C,D**) MIF expression in MN patients.

**Figure 5:**
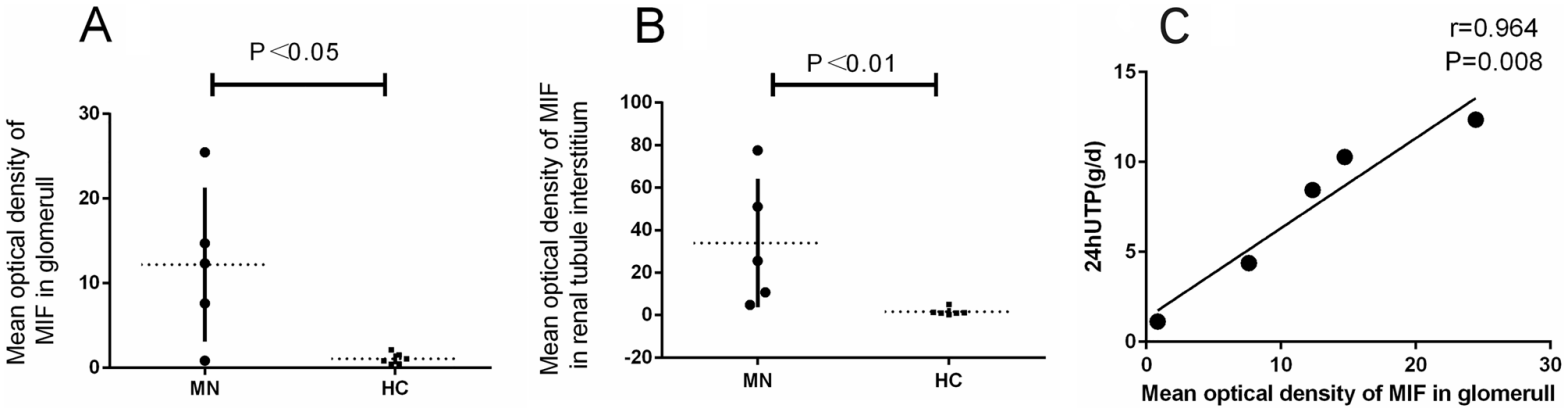
Comparison of MIF expression levels of kidney tissues in MN patients and controls. (**A**) Comparison of MIF expression levels in the glomeruli between MN patients and controls, *P* < 0.05. (**B**) Comparison of MIF expression levels in the tubular stromal region of MN patients and controls, *P* < 0.01. (**C**) Correlation between glomerular MIF expression and UTP/24 h: glomerular MIF expression in MN patients (r = 0.964, *P* = 0.008).

The renal histopathology of MN patients was also studied. In the renal specimens of MN patients, immunohistochemical staining revealed prominent expression of MIF in glomerular basement membrane and the renal interstitium (Fig. 4). Compared with control group, the mean optical density of MIF in MN patients in glomeruli was significantly higher (12.19 ± 9.11 vs. 1.05 ± 0.66, *P* = 0.015) (Table 4). The mean optical density in tubular stromal MIF in MN group compared with control group (*P* = 0.009) was statistically significant (Fig. 5A, B). Among the MN patients, correlation analysis suggested that the mean optical density of MIF-positive was correlated with levels of 24-h UTP (*P* = 0.008) (Fig. 5C).

**Table 4:** Level of MIF expression in the renal tissue.

	MN (n = 5)	Control (n = 6)	*P*
Mean density of the glomerular area	12.19 ± 9.11	1.05 ± 0.66	0.015
Mean density of the renal tubular interstitial area	25.51 (7.79, 64.30)	1.12 (0.86, 2.19)	0.009

## Discussion

MIF is an inflammatory cytokine with properties of chemokines that it initiates host immune responses^15^. MIF could be a therapeutic target in a number of immune-related such as asthma^16^, rheumatoid arthritis^17^, ulcerative colitis^18^, systemic sclerosis^19^, and lupus erythematosus^20^. Overall, MIF regulates cytokine expression, promotes inflammatory cell recruitment, and triggers and amplifies the effects of other proinflammatory cytokines, as well as its glucocorticoids-opposing effect^21,22^. Angiotensin II may promote MIF synthesis and secretion in renal tubular epithelial cells, leading to mediate renal injury^23^. A number of scholars have found that the urinary MIF concentration significantly increases in proliferative glomerulonephritis, and urinary MIF level reflects MIF expression within the kidney in crescentic GN, particularly in disease exacerbation^24^. Furthermore, a previous study demonstrated that urinary excretion of MIF could be a prognostic marker only in proliferative glomerulonephritis, accompanying with higher values related to a worse prognosis^25^. Increased MIF locally produced and secreted in damaged kidneys could be associated with a progressive increased production of MIF in proliferative glomerulonephritis, according to the immunohistochemical staining and measurement of urinary MIF concentrations. However, the same statistically significant relationship was not found in the group of non-proliferative glomerulonephritis, which could be related to the lower rate of MIF excretion in these diseases. This is consistent with our findings that the difference in plasma and urinary MIF levels between MN and control groups was not statistically significant, and we analyzed the differences in MIF levels after Cr correction between these two groups. Additionally, it was revealed that the plasma and urinary MIF levels gradually increased with the elevated severity of renal pathological damage in MN patients.

MN pathogenesis is complex, the high incidence of MN has been reported in recent years, and kidney biopsy is the gold standard for its diagnosis. However, because kidney biopsy is accompanied with a number of complications, some patients decline undergoing kidney biopsy, and several scholars aim to find out less risky diagnostic methods for MN using biomarkers. Beck et al. first reported M-type phospholipase A2 receptor (PLA2R) as the major membranous nephropathy target antigen, and testing of 70–80% of patients with primary MN for serum anti-PLA2R antibodies was positive^3^. We, in the present study, analyzed the MIF levels in plasma and urine between the two groups of MN patients with positive and negative PLA2R-Ab expressions, and we did not find consistency of PLA2R-Ab expressions with MIF levels.

MIF expression is typically associated with infection or pathogenic inflammatory conditions^26^. For instance, bacterial infections, including the most severe endotoxin shock cases, are associated with the high MIF concentrations produced by macrophages during immune cell-mediated bacterial clearance^27^. Therefore, we hypothesized that the occurrence of infection in MN patients was correlated with the MIF levels, and we analyzed the correlation of plasma and urinary MIF levels in MN patients with infection-related clinical indicators. The main receptor of MIF is CD74, and it can bind to CD44 to form a receptor complex and mediate MIF signaling transduction, while CD74 can also form complexes with CXCR2 and CXCR4 to deliver MIF signaling to integrins in inflammatory cells^28–30^. After triggering inflammation, high MIF concentrations may be fatal due to uncontrolled initiation of stromal cytokines. Therefore, the rescue of mice from lethal endotoxemia by MIF deletion or application of anti-MIF antibodies could not be advantageous. The beneficial effects of anti-MIF treatment were achieved even when anti-MIF treatment was applied after the onset of infection^31^. Thus, MIF mainly plays an upstream role in the inflammatory cascade due to its inflammation-inducing activity.

Patients with MN are at an extremely high risk of concurrent thromboembolism, which is the most common and severe complication^32,33^. Experimental in vitro and in vivo studies have found that MIF interaction with CXCR7 modulates platelet survival and thrombotic potential^34^. In the present study, the correlations of plasma and urinary MIF levels and prognostic indicators associated with coagulation abnormalities in MN patients were analyzed, and it was found that MIF levels were associated with the hypercoagulable status in MN patients. It was reported that human platelets do not only contain significant amounts of MIF protein, but also are able to secrete MIF upon specific thrombogenic stimulation^35^. Tadamichi et al. found that MIF cooperates with thrombin at the site of injury to promote wound healing, thrombin induces MIF mRNA expression in endothelial cells, and this expression can be specifically blocked by the thrombin-specific inhibitor hirudin^36^.

Due to the low rate of MIF excretion in the kidney, numerous scholars attempted to explore the MIF expression in the kidney tissue. The results of a previous study showed that tubular epithelial MIF expression was significantly upregulated in renal puncture pathological tissue of patients with IgA nephropathy, and hyperplastic mesangial cells also expressed MIF^37^. In experimental rat glomerulonephritis, renal MIF protein expression was markedly upregulated, which was associated with an increase in the number of MIF-positive podocytes in the rat diseased glomerular against Thy1 nephritis^38^. Some scholars demonstrated the expression of MIF protein in kidney tissue of human proliferative glomerulonephritis, and this was correlated with leukocyte infiltration, histologic damage, and renal function impairment^39^. In addition, Vincenzo et al.^40^ applied matrix-assisted laser desorption/ionization mass spectrometry imaging (MALDI-MSI) to analyze two homogeneous groups of patients with a renal biopsy that confirmed stage MN-II, and then, their responses were observed and followed up. They finally found that MIF could be used as a biomarker to distinguish which patients deferentially responded to the immunosuppressive treatments. This finding verified the putative predictive role of MIF in terms of outcome and response to standard therapy of MN patients, and also supported prognostic assessment of MN patients. The current study not only confirmed that MIF was expressed in the kidney tissues of MN patients, but also this expression was found to be associated with 24-h UTP (Supplementary Information).

In conclusion, plasma and urinary MIF levels could reflect the severity of MN, and they could be associated with venous thrombosis and infectious complications in MN patients. Besides, MIF was significantly expressed in the renal tissue of MN patients, and glomerular MIF expression could be used to indicate the disease activity.

## Supplementary Information

Supplementary Information.

## Data Availability

The datasets used and/or analyzed during the current study are available from the corresponding author on reasonable request.
