# Supplementary material for: Macrophage migration inhibitory factor levels are associated with disease activity and possible complications in membranous nephropathy
Source: Sci Rep. 2022 Nov 3;12:18558. doi: 10.1038/s41598-022-23440-1 (PMC9633699; doi:10.1038/s41598-022-23440-1)
Supplement: Supplementary file 1 — Supplementary Information. [file 41598_2022_23440_MOESM1_ESM.pdf]

Supplementary Data

Title **Macrophage migration inhibitory factor levels are associated with disease activity and possible complications in membranous nephropathy**

Authors **Na Ding[b]; Peng-Lei Li[a]; Kai-Li Wu[b]; Tie-Gang Lv[a]; Wen-Lu Yu[ab]; Jian Hao，MD[ab*]**

Table S1 Infection patients with MN

Type of infection	number
Urinary tract infection	4
Upper respiratory tract infection	3
Acute attack of chronic tonsillitis	1
Amount	8

Table S2 Thrombus formation patients with MN

Type of Thrombus formation	nmuber
Intermuscular vein thrombosis in the left calf	3
Interamuscular vein thrombosis in the right calf	4
Deep venous thrombosis in the lower limbs	3
Acute cerebral infarction	2
Amount	12

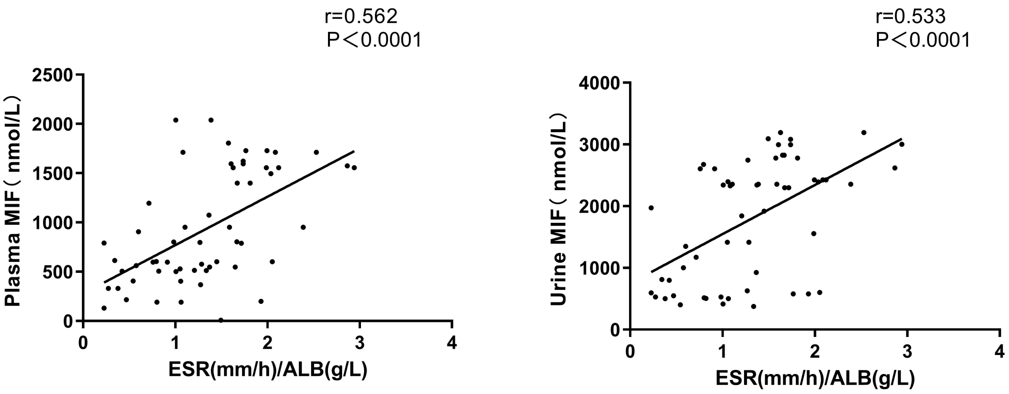

Figure S1. MIF levels in plasma (A) and urinary (B) were positively correlated with ESR/Albumin ratio indicators

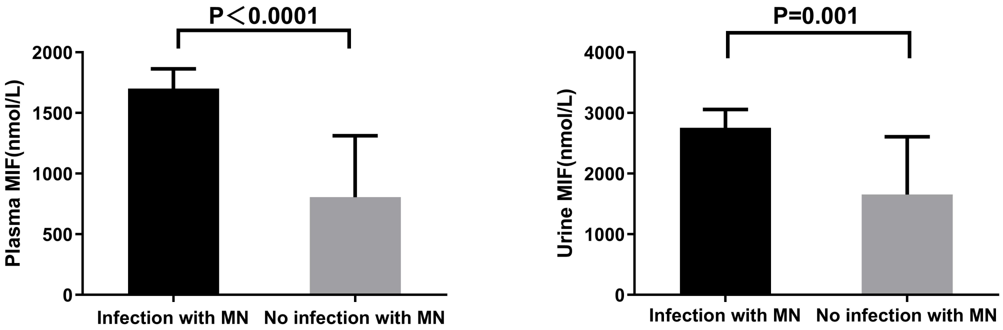

Figure S2. MIF levels in plasma (A) and urinary (B)
